# Novel Clinical Practice Assessments: Informational Statements by the Musculoskeletal Tumor Society

**DOI:** 10.1002/jso.27967

**Published:** 2024-10-28

**Authors:** Nicholas S. Tedesco, Matthew Wallace, Yee‐Cheen Doung, Matthew Colman, Felasfa Wodajo

**Affiliations:** ^1^ Department of Orthopedic Surgery WUCOM‐PNW, Good Samaritan Regional Medical Center Corvallis Oregon USA; ^2^ Surgery and Perioperative Care University of Texas Dell Medical School Austin Texas USA; ^3^ Department of Orthopaedics and Rehabilitation Oregon Health & Science University Portland Oregon USA; ^4^ Department of Orthopaedic Surgery Rush University Medical Center Chicago Illinois USA; ^5^ Department of Orthopaedic Surgery University of Virginia School of Medicine ‐ Inova Campus, Virginia Cancer Specialists Fairfax Virginia USA

**Keywords:** clinical practice, evidence‐based, MSTS, novel, review

## Abstract

Musculoskeletal oncology involves rare diseases. As a result, there is a paucity of literature to guide practitioners. Studies are often clinical experience, retrospective reviews, noncomparative studies, and involve small numbers of patients. However, technological advances consistently arise in this field. This article represents the Musculoskeletal Tumor Society efforts to improve multispecialty collaboration and research credibility. It involves brief systematic reviews of novel ideas and suggests high‐quality research needed to provide and standardize best practices within this field.

## 1 Introduction

The United States Federal Government had a total of $179.5 billion in scientific research and development funding obligations in 2021 [1]. The Department of Health and Human Services (DHHS) trailed only the Department of Defense (DOD) in earmarked dollars. The National Institute of Health (NIH) falls under the purview of DHHS, and the National Cancer Institute received more funding than any other NIH division [2]. Additionally, several DOD projects were aimed at the current presidential priority of cutting cancer deaths in half [1], making cancer research one of the most heavily funded scientific disciplines in the United States today. As a result, cancer research and technology continue to be some of the most burgeoning and rapidly changing scientific disciplines from year to year.

The ever‐growing complexity of cancer care continues to warrant a multidisciplinary approach to optimize patient outcomes [3]. Although the optimal methods for incorporating multidisciplinary care into clinical practice are yet to be elucidated, the largest treatment roles continue to center around surgical management, procedural management, medical oncology, and radiation oncology. As a result of the rapidly changing landscape of cancer care, coupled with the rarity of musculoskeletal tumors, the Musculoskeletal Tumor Society (MSTS) has leveraged their Guidelines and Evidence‐Based Medicine (GEBM) committee to produce the following novel clinical practice assessments based on systematic reviews of each topic. Further information on the topics and methods of data extrapolation can be found on the MSTS website at https://www.msts.org/index.php/education/novel-clinical.

These informational statements are an evidence‐based review of some of the most prescient and growing topics in musculoskeletal oncology with no formal recommendations or guidelines. Rather, they are meant to be a comprehensive literature review of the available literature of new and emerging technologies within the multidisciplinary fields involved with caring for benign and malignant musculoskeletal system tumors. The goal is to provide a succinct conglomeration of evidence to allow practitioners to decide if or how they may incorporate these ideas into their practice, and make calls for high‐quality, future research to help to continue to guide us in an effort to standardize optimal treatment strategies throughout the United States. As evidence is expected to change rapidly, these diagnostics and treatments should serve as starting points for clinical practice and clinical decision aids during discussions and informed decision‐making with patients.

The authors have the following potential conflicts of interest to disclose: Nicholas Tedesco owns private stock in Doctorpedia and ROMTech Inc., is on the editorial board of the Journal of the American Osteopathic Academy of Orthopedics, received publishing royalties from Sanara MedTech, is a committee member for the Musculoskeletal Tumor Society and American Academy of Orthopedic Surgeons. Matthew Wallace has nothing to disclose. Yee‐Cheen Doung is a committee member for the Musculoskeletal Tumor Society. Matthew Colman is a paid consultant for Alphatec Spine, DuPuy, K2M/Stryker Spine, CSRS, Orthofix, Spinal Elements, and Xenix Medical. He also is a committee member for the AO Spine North America, Cervical Spine Research Society, Musculoskeletal Tumor Society, LSRS, and the North American Spine Society. Finally, Felasfa Wodajo is a paid consultant for Onkos Surgical and is a committee member for the Musculoskeletal Tumor Society.

## 2 Pexidartinib and the ENLIVEN Clinical Trial

### 2.1 Background

Tenosynovial giant cell tumor (TSGCT, formerly “pigmented villonodular synovitis”) is a benign synovial neoplasm mixed with inflammatory response. Only a minority of the neoplastic cells express CSF‐1 as a result of genomic aberrations on chromosome 1p13. However, this overexpression recruits numerous monocytes, macrophages, and other inflammatory cells to the milleu [4]. This overexpression of CSF‐1 has been shown to contribute to pain, swelling, and tumor evolution in patients afflicted with this disease. It can exist as a more indolent solitary nodule (localized or nodular) or involve the entire tenosynovium of the afflicted joints/tendons that exhibits a more locally aggressive behavior (diffuse) [5].

### 2.2 What's New?

Pexidartinib (Turalio, Daiichi Sankyo Inc., Basking Ridge, NJ) is a novel small molecule immune checkpoint tyrosine kinase inhibitor with selective activity against the CSF‐1 receptor (CSF‐1R). Its proof‐of‐concept, efficacy, and safety have been developed through their ENLIVEN clinical trial with an independent data monitoring committee responsible for patient safety and overseeing the study (Table 1) [6].

**Table 1:** Summary of the current Musculoskeletal Tumor Society novel clinical practice assessment.

Technology	Evidence in favor	Evidence against	Deficiencies in the available data
Pexidartinib in TSGCT	Nonsurgical, non‐radiation option for patients that have failed surgical treatment or are not surgical candidates, and good results in terms of symptom reduction and tumor regression	Some trial patients experienced severe hepatotoxicity and mortality, treating providers need to be enrolled in manufacturer's REMS program to prescribe, and very high cost of treatment	Unclear optimal dosage, how long to continue therapy, or if and how it should be done in conjunction with surgery
3‐D PSI in bone sarcoma sx	Precision bone resection can be achieved, can be used with matched allograft or 3D‐printed custom implant reconstructions, and facilitates surgery for complex anatomy such as pelvis/spine	Additional expense and difficulty with insurance coverage, and time for fabrication must be planned to avoid risk of delaying treatment	Extensive bias in existing studies, limited comparative data, many studies show the theoretic benefits of accurate bone cuts, but unknown if this actually improves oncologic or functional outcomes
Doxycycline injections for ABC's	Injection is minimally invasive, material is easy to obtain and affordable, and appears to have similar or improved recurrence rates as compared to surgery	May need multiple injections and long time intervals of treatment to adequately treat tumor, and complications include skin necrosis and local recurrence	Extensive bias in existing studies, limited comparative data, incomplete reporting of data, short follow‐up and small numbers in case series
Radiolucent implants in skeletal oncologic sx	Improved visibility of anatomy on advanced imaging, closer modulus of elasticity to bone, improved fatigue strength, and improved ability to irradiate local tissues	Availability and cost, workability of implants more difficult due to inability to bend/contour, and implant position or failure may be difficult to visualize	Many studies show the theoretic benefits of better imaging of local anatomy and ability to deliver better radiation, but unknown if this actually improves oncologic or functional outcomes, and optimal imaging modality to assess for implant failure still unknown
Percutaneous ablation for skeletal metastases	Single treatment with rapid onset of pain relief, can be performed concurrently with percutaneous stabilization via cementoplasty when needed, can be used when patients cannot receive further radiation and low reported complication rates	Inadequate evidence comparing efficacy and durability versus radiation therapy, inadequate reporting of bone structural integrity and fracture risk, and requires anesthesia to be performed	No prospective comparative studies that include ablation versus radiation versus ablation with radiation, optimal ablative modality unknown

Abbreviations: ABC, aneurysmal bone cyst; PSI, patient‐specific instrumentation; REMS, risk evaluation and mitigation strategy; sx, surgery; TSGCT, tenosynovial giant cell tumor.

### 2.3 Discussion

The ENLIVEN Clinical trial was a Phase III, open label, double blind, placebo controlled, clinical trial investigating the safety and efficacy of pexidartinib (Turalio) for the treatment of symptomatic, refractory or recurrent, and unresectable diffuse tenosynovial giant cell tumor (D‐TSGCT) [6]. Final enrollment included 120 patients (61 receiving pexidartinib and 59 receiving placebo). There were no significant demographic differences between groups, and the knee was the most common anatomic site. Nine patients receiving pexidartinib and 11 in the placebo group withdrew from the study due to adverse events.

Overall response rate by RECIST was 39% versus 0% in the pexidartinib versus placebo groups, and all responses were maintained out to 6 months of follow‐up. Range of motion, PROMIS functional outcome scores, stiffness, and pain were all also improved in the pexidartinib group over placebo and correlated with RECIST response. However, 30 patients crossed over from the placebo to pexidartinib group. Additionally, those allocated to the placebo group were allowed to receive the drug after 6 months [6].

Continued follow‐up of patients in the ENLIVEN trial over several years have demonstrated lasting and improved response rates compared to those seen during the active trial [7]. Because of the hepatotoxicity seen in the trial at higher doses along with subsequent noninferiority studies, current FDA‐approved treatment recommendations for pexidartinib are an oral 400 mg tablet, twice daily (800 mg/d), or 400 mg in the morning and 200 mg in the evening (600 mg/d) in patients with renal impairment [7].

However, Grade 3 or 4 adverse events (AEs) occurred in 27 (44%) and 7 (12%) patients receiving pexidartinib or placebo, respectively. The AEs that occurred more frequently in the pexidartinib group and included skin and hair hypopigmentation (61% vs. 3%) and elevations in alanine transaminase, aspartate aminotransferase, and alkaline phosphatase (7%–10% vs. 0%). Seven patients discontinued pexidartinib due to mixed and cholestatic liver toxicity. Most of the hepatotoxicity was reversible within 1–2 months of stopping pexidartinib [6]. Serious hepatotoxicity emerges within the first 2 months of treatment that warrants aggressive liver function tests monitoring early during administration. Pexidartinib should be avoided in patients with pre‐existing liver or biliary tract diseases and in patients with elevated bilirubin or transaminases at baseline [7]. Because of the black box warning regarding the risk of potentially fatal liver injury, it is prescribed and dispensed solely via a manufacturer‐supported Risk Evaluation and Mitigation Strategy safety program [8].

Findings from animal studies suggest pexidartinib may impair both male and female fertility, and is a potential teratogen to a developing fetus. Therefore, in women of child‐bearing age, pregnancy status must be verified with adequate contraception used during the course of treatment and 1 week after the final dose. Similarly, lactating women should not breastfeed during the course of treatment and 1 week after the final dose [8].

### 2.4 Future Directions

Questions that remain to be answered with future research are precise indications for the use pexidartinib, pexidartinib as monotherapy versus adjuvant to surgery, optimal dosing, optimal duration of treatment, and safety and efficacy in children. Other small molecule CSF‐1R inhibitors currently being developed or investigated include ARRY‐382 (Array BioPharma), PLX7486 (Plexxikon), BLZ945 (Novartis), and JNJ‐40346527 (Johnson& Johnson). Current anti‐CSF‐1R mAbs include emactuzumab (Roche), AMG820 (Amgen), IMC‐CS4 (also referred to as LY3022855; Eli Lilly), cabiralizumab (Five Prime Therapeutics), MCS110 (Novartis; CSF‐1), PD‐0360324 (Pfizer, CSF‐1) [9], and the intraarticular CSF1R inhibitor, AMB‐05X (AmMax Bio Inc.) [10].

## 3 Three‐Dimensionally (3D)‐Printed Patient‐Specific Instruments in Bone Sarcoma Surgery

### 3.1 Background

Limb‐salvage, margin‐negative surgery is the mainstay of treatment for sarcomas of bone. The musculoskeletal oncology surgeon is tasked with optimizing the long‐term functional outcomes of the patient while maintaining appropriate oncologic outcomes. Free‐hand resections of bone tumors are associated with discrepancies between planned resection and specimen lengths up to 20 mm, with an unplanned intralesional margin in 8% of cases [11].

### 3.2 What's New?

Recent advances in tumor imaging and 3D technologies may permit more accurate, tighter resections around bone sarcomas that facilitate limb and joint salvage and/or reconstruction (Table 1). 3D technology may be used to create surgical models that help visualize surgery and measure resections preoperatively, and intraoperative 3D navigation has been used with computed tomography (CT)‐based optical navigation systems to localize and direct instruments in real time. Currently, patient‐specific instruments (PSI) and implants can be designed and fabricated, which are then applied onto or inserted into the bone to precisely template and complete bony cuts, size‐match allografts, or provide custom replacements for reconstruction. However, these advances come with both time and monetary expenses. The clinical benefits of PSI are still under scrutiny as emerging practices.

### 3.3 Discussion

Only one study provided a case‐control series comparing PSI resection to manual free‐hand resections [12]. The other five are cohort studies that evaluated between 6 and 31 patients [11, 12, 13, 14, 15, 16]. There was only one reported intralesional osteotomy with PSI (1%) [12]. There were two planned R1 margins adjacent to critical structures (2%) [13, 14], and all other osteotomy margins were negative (97%). Soft tissue margins were infrequently reported but none were reported as positive. The difference between the planned resection and the measured specimen was low, generally 0–3 mm [13, 15]. These differences may be partially accounted for by the thickness of the surgical saw blade. Complications rates are generally attributed to the procedure and method of reconstruction rather than the use of PSI, which was not found to be consistently associated with differences in operative time or blood loss.

### 3.4 Future Directions

There is much information needed to evaluate the use of patient‐specific instrumentation. PSI may be of benefit in obtaining negative margins in bone sarcoma surgery, but it remains to be seen if PSI‐matched allografts or custom implants made with solid free‐form fabrication or additive manufacturing demonstrate better long‐term functional outcomes.

## 4 Percutaneous Doxycycline Injection for Aneurysmal Bone Cysts

### 4.1 Background

Primary aneurysmal bone cysts (ABC's) are a benign, but locally aggressive and destructive bone tumor, most commonly affecting individuals in the second decade of life. Local recurrence rates after surgical curettage vary from 10% to 70% with higher risk of recurrence seen in children < 10 and juxtaphyseal lesions, probably from less aggressive management aimed at sparing growth plates. It can involve any bone in the body, but in the spine and pelvis, it is particularly difficult to adequately treat given the critical nearby critical anatomic structures [17].

### 4.2 What's New?

Doxycycline is commonly used as an antibiotic. However, it has also been found to heal lymphatic malformations that are associated with overproduction of vascular endothelial growth factor and matrix metalloproteinases (MMPs). In cell culture, doxycycline has been shown to inhibit MMP, angiogenesis, and osteoclastic function. Therefore, it has been proposed to be toxic and effective at inducing regression of some of the driving mechanisms of the local destruction in ABC's. A doxycycline and albumin protein foam is an injectable substance that can be targeted to aneurysmal bone cysts (Table 1) [18].

### 4.3 Discussion

The earliest documentation of this technique was in 2013 [18]. Currently, there have been seven studies reported in the literature [18, 19, 20, 21, 22, 23, 24]. All seven were retrospective and involved percutaneous injection of doxycycline into Primary ABC's. Of these studies, one had a comparison cohort [21]; the remaining six were case series. Three studies were limited to the spine [20, 21, 23]. The number of cases treated with doxycycline in each series ranged from 7 to 21 with the total number of doxycycline injections per patient ranging from 1 to 15. The rate of recurrence varied between 0% and 14%. In the single comparison study, 14 patients were treated with doxycycline alongside 11 patients who received surgery. There were no recurrences in the group treated with doxycycline compared to two in the group treated with surgery; however, this difference was not statistically significant (*p* = 0.14) [21].

The follow‐up for these studies is varied and is as short as 4 months [19]. While data is promising for decreased local recurrence rates, for the patients whose follow‐up is less than 24 months, it is difficult to assess. Furthermore, the total number of cases involved in each study is fewer than 25. There are two reported incidences of skin necrosis from the injection. In addition, most patients are treated with a median of 3–5 treatments. For pediatric patients, this likely means 3–5 anesthetic sedations.

### 4.4 Future Directions

At this time, there is only one case–control study and no prospective cohort studies looking at the effectiveness of percutaneous doxycycline injection for ABC's. The early evidence for doxycycline is promising but the total data is limited and even more limited in comparison to surgery. Furthermore, it is unclear how many treatments over how long of a time period are needed for adequate control of disease. A multicenter clinical trial will ultimately be needed to determine cost, time involved, optimal patient experience, safety, and efficacy of doxycycline injections for ABC's as compared to surgery as the historic standard.

## 5 Use of Radiolucent Implants in Surgery for Musculoskeletal Oncology

### 5.1 Background

The treatment of metastatic bone disease or primary tumors in the axial or appendicular skeleton frequently requires reconstruction or stabilization to maintain or restore functional capacity. Traditional metallic implants are the gold standard but may negatively impact postoperative imaging and radiotherapy plans as a result of artifactual scatter or voids on advanced imaging and radiation diffraction, respectively. Additionally, patients requiring proton beam radiation frequently must have metallic implants removed to facilitate planning and delivery of accurate dosing.

### 5.2 What's New?

A variety of radiolucent carbon‐fiber reinforced implants have emerged for the reconstruction and stabilization of defects in both the spine and extremities. The rationale for use of these implants involves the ability to obtain immediate postoperative imaging without reduction in image quality, the ability to deliver radiation accurately without beam perturbation, modulus of elasticity closer to native bone than metal, and an improved fatigue strength profile (Table 1).

### 5.3 Discussion

In the spine, several clinical studies have detailed the surgical efficacy, clinical outcomes and technical challenges of radiolucent implants in primary and metastatic *spinal* tumors. Generally speaking, the studies were small and underpowered to show non‐inferiority. Nevertheless, most studies claim similar clinical outcomes and perioperative complications when comparing carbon implants to traditional titanium implants. Most studies highlight the anecdotal and non‐quantified benefits of improved postoperative imaging and more easily facilitated radiation planning.

For instance, one study highlighted that in their series they detected three early recurrences out of a total 28 cases thanks to the better imaging characteristics of the carbon system [25]. Other studies have assessed perturbation of radiation delivery for carbon implants in comparison to traditional titanium implants. One study showed a greater than 30% reduction in the difference between plan and actual delivery of photons with carbon implants [26]. Laux et al. [27] whose another study compared photons to protons using carbon or metallic implants, and showed nearly twice the deviation from plan with proton delivery around metallic implants in comparison to photons, highlighting consideration of presence of metallic implants with the use of protons specifically.

In the extremities, two clinical studies have compared carbon intramedullary nails to titanium implants and found no complication or clinical outcome differences between the groups. No nail breakage events occurred, and biomechanical strength of carbon implants was highlighted [28, 29].

Two studies have illustrated the absence of artifacts and optimal visualization of bone and soft tissues on postoperative CT and magnetic resonance imaging (MRI) [30, 31]. However, one study using carbon‐fiber‐reinforced PEEK intramedullary nails noted a 13% intraoperative and 7% postoperative complication rate, with radiographic union in only 14/53 patients [30].

### 5.4 Future Directions

Carbon implants may offer advantages over traditional implants in terms of postoperative MRI and CT imaging, as well as accurate delivery of radiation plans. While no clear inferiority has been established with regard to clinical outcomes and complications, concerns have been raised regarding implant visualization intraoperatively and implant system workability. Therefore, further clinical study and instrumentation evolution should occur before widespread adoption.

## 6 Percutaneous Ablation for Skeletal Metastases

### 6.1 Background

The majority of patients with carcinomas will eventually develop skeletal metastases. In many cases, these may be painful and, in some cases, lead to fracture. The primary modality for alleviation of pain from skeletal metastases is external beam radiation. However, some patients may have failed radiation therapy and/or have mechanical instability at the site of metastasis requiring treatment. In these cases, other modalities to address the symptoms associated with skeletal metastatic disease may be indicated.

### 6.2 What's New?

Percutaneous radiofrequency ablation (RFA) is a minimally invasive procedure that be done in the outpatient setting and has been shown to be effective in reducing pain from skeletal metastases (Table 1). RFA is not new, but there has recently been a more vigorous interest in expanding its utilization for skeletal metastases. Thus far, the bulk of the evidence is in the spine, but there are increasing reports of its successful use in the pelvis and extremities.

### 6.3 Discussion

Two studies have compared the outcomes of RFA alone versus RFA with cementoplasty for local tumor control and/or tumor‐related pain [32, 33]. Both were successful with no differences in outcomes or complications. However, bone structural integrity was not specifically addressed.

Most studies of non‐spinal metastases tend to report on lesions in the sacrum or pelvis with very few studies looking at extremity metastases. Other forms of percutaneous ablative therapy have also been studied, including cryoablation (CTx) and microwave ablation (MWA). One notable study demonstrated better outcomes and pain control with propensity matching patients undergoing cryoablation versus radiofrequency ablation [34]. Outcomes are mostly promising, but there is wide heterogeneity in histology, additional treatments, location of treatment, and the prognosis of patients. Additionally, most reports are case series and cohort studies with very few comparative studies. As a result, optimal indications and ablative modality is yet to be elucidated.

### 6.4 Future Directions

Separate prospective studies randomizing patients with symptomatic spinal bone metastases and extremity metastases to percutaneous ablative treatment (RFA, MWA, or CTx) versus conventional external beam radiation should be feasible and would be very helpful in making treatment recommendations. It could also help provide comparative cost data, patient reported outcome measures, convenience (one ablative treatment vs. multiple radiation treatments) measures, and time to symptomatic improvement. Additionally, subgroup analysis should be done to determine success rates in terms of specific tumor locations as well as structural integrity of the remaining bone, using either descriptive (i.e., % of cortical destruction) or fracture risk prediction modeling (i.e., Mirel's score [35] or CT structural rigidity analysis [36]).

## 7 Summary

The landscape of musculoskeletal oncology care is changing rapidly. This article addresses the available literature of several emerging technologies that are steadily gaining acceptance and interest throughout the United States. Further research will be needed on all of these topics to define their use and efficacy, but early results appear to be very promising for all of them.

Pexidartinib is a promising nonsurgical option for patients debilitated by diffuse tenosynovial giant cell tumor. However, indications, dosage, long‐term efficacy and risk, role in combination with surgery, and duration of use have yet to be defined. 3D printed patient‐specific instrumentation for complicated and accurate osteotomies in bone sarcoma management to improve the ability to provide limb‐salvage, margin‐negative surgery and reproduce accurate planes for custom allograft or implant fixation. However, cost, insurance coverage, indications, and whether or not they actually improve oncologic and surgical outcomes remains to be seen. Percutaneous doxycycline injections for aneurysmal bone cysts have the potential to reproduce similar cure rates to surgery while being far less invasive and mutilating. However, this technology is in its infancy. These are technically challenging procedures requiring highly skilled interventional radiologists, and often require several series of injections over several months to years. Therefore, it remains to be seen how effective it truly is given the extensive bias and methodological flaws in the available literature, as well as how practical it will be for universal adoption. Carbon fiber implants for structural support and fixation in the spine and extremities offer several advantages over traditional titanium or stainless steel implants including improved visibility of local anatomy on advanced imaging, closer modulus of elasticity to bone, and improved radiation diffraction coefficients. However, cost, availability, specific indications, streamlined insertion instrumentation, and long‐term failure rates remain to be defined. Finally, similar to doxycycline in ABC's, RFA for symptomatic, but structurally stable carcinoma skeletal metastases offers a minimally invasive potential for treatment while obviating the side effects and multiple treatments needed with radiation or the risks and recovery of surgery. However, the optimal ablative modality (RFA, MWA, or CTx) in terms of cost, efficacy, and risk has been undefined. Further, risk of fracture pretreatment or posttreatment given the kill zone of the ablative technique reaching the otherwise intact bony cortex both remain to be taken into account or reported in available studies.

## Ethics Statement

This study was conducted in aggregate through the Guidelines and Evidence‐Based Medicine Committee for the Musculoskeletal Tumor Society. It was written, performed, and edited at each author's respective institutions independently.

## Conflicts of Interest

Nicholas Tedesco – Doctorpedia: Stock or stock Options. Journal of the American Osteopathic Academy of Orthopedics: Editorial board. Sanara MedTech: Publishing royalties, financial or material support. Musculoskeletal Tumor Society: Board or committee member. American Academy of Orthopedic Surgeons: Board or committee member. RomTech Inc.: Stock or stock Options. Matthew Wallace – Nothing to disclose. Yee‐Cheen Doung – Musculoskeletal Tumor Society: Board or committee member. Matthew Colman – Alphatec Spine: IP royalties; Paid consultant. AO Spine North America: Board or committee member; Research support. Cervical Spine Research Society: Board or committee member. Musculoskeletal Tumor Society: Board or committee member. CSRS: Research support. DePuy, A Johnson & Johnson Company: Paid presenter or speaker. K2M: Paid presenter or speaker. K2M/Stryker Spine: Paid consultant. LSRS: Board or committee member. Musculoskeletal Tumor Society: Board or committee member. North American Spine Society: Board or committee member. Orthofix: Paid consultant. Orthofix Inc.: Paid presenter or speaker. Spinal Elements: IP royalties; Paid consultant. Xenix Medical: Paid consultant. Felasfa Wodajo – Onkos Surgical: Paid consultant. Musculoskeletal Tumor Society: Board or committee member.

## Synopsis

The landscape of musculoskeletal oncology care is changing rapidly. This article addresses the available literature of several emerging technologies that are steadily gaining acceptance and interest throughout the United States. Further research will be needed on all of these topics to define their use and efficacy, but early results appear to be very promising for all of them.

## Data Availability

Data available upon request from the authors.
